# Retraction Note: miR-195-5p/NOTCH2-mediated EMT modulates IL-4 secretion in colorectal cancer to affect M2-like TAM polarization

**DOI:** 10.1186/s13045-023-01438-0

**Published:** 2023-04-19

**Authors:** Xiaobin Lin, Shuyi Wang, Min Sun, Chunxiao Zhang, Chen Wei, Chaogang Yang, Rongzhang Dou, Qing Liu, Bin Xiong

**Affiliations:** 1 Department of Gastrointestinal Surgery, Zhongnan Hospital of Wuhan University, No.169 Donghu Road, Wuchang District, 430071 Wuhan China; 2 Department of Gastric and Colorectal Surgical Oncology, Zhongnan Hospital of Wuhan University, No.169 Donghu Road, Wuchang District, 430071 Wuhan China; 3 Hubei Key Laboratory of Tumor Biological Behaviors, No.169 Donghu Road, Wuchang District, 430071 Wuhan China; 4 Hubei Cancer Clinical Study Center, No.169 Donghu Road, Wuchang District, 430071 Wuhan China; 5 Department of General Surgery, Taihe Hospital, Hubei University of Medicine, Shiyan, 442000 China

**Correction to: Journal of Hematology & Oncology (2019) 12:20** https://doi.org/10.1186/s13045-019-0708-7

The Editor in Chief has retracted this article. After publication, concerns were raised in relation to overlaps between Figs. 2G and 4G as well as 2G and 4E. Additional investigation by the publisher found further concerns:

- Further duplicated images have been found in Fig S6D with different data labels;
- In Fig. S6H an image of mice appears to not interact with the background in Fig. S6H.

Therefore, the Editor in Chief has lost confidence in the integrity of the results. Bin Xiong has stated on behalf of all authors that they disagree to this retraction.

